# The Ear, the Heart, and the Lung: The Mighty Mite

**DOI:** 10.7759/cureus.77658

**Published:** 2025-01-19

**Authors:** Divya Joyce Susai Manickam, Umarani Ravichandran

**Affiliations:** 1 Internal Medicine, Sri Ramachandra Institute of Higher Education and Research, Chennai, IND; 2 Internal Medicine, Government Medical College & Hospital Cuddalore, Chidambaram, IND

**Keywords:** eschar, infection and arrhythmia, infection-related hearing loss, scrub typhus, tropical infections

## Abstract

Scrub typhus is a common cause of febrile illness. It is a major public health threat in the Asia-Pacific region, particularly the “Tsutsugamushi triangle” geographic region. Patients present with fever, rash, nausea, and vomiting, and may later develop systemic manifestations. The characteristic and typical “eschar” of the disease might be absent, making the diagnosis difficult. This case report highlights the importance of recognizing uncommon clinical presentations of this common tropical disease and its prompt diagnosis and treatment.

## Introduction

Scrub typhus is caused by *Orientia tsutsugamushi*, a gram-negative bacteria, and is transmitted by the bite of the larvae/chigger of trombiculid mites. It is a common infection in South and Southeast Asia [1]. These mites reside among vegetation and are maintained among rodents [2]. Humans are thus accidental hosts. The mortality ranges from between 30% to 70% if not identified and promptly treated [3].

After the bite by the vector, there is a relatively symptom-free period of six days to 21 days, followed by a clinical spectrum of manifestations secondary to systemic vasculitis [4]. Initially, a painless papule appears, which later ulcerates and evolves into a black-crusted “eschar.” Then there is a symptomatic phase characterized by fever, rash, myalgia, nausea, vomiting, lymphadenopathy, and conjunctival suffusion [5]. Complications including jaundice, meningoencephalitis, interstitial pneumonia, renal failure, acute respiratory distress syndrome and multiorgan dysfunction syndrome (MODS) are well established [5,6]. The diagnosis is made by using tests such as immunofluorescent assay and indirect immunoperoxidase, which are considered the gold standard, and also by enzyme-linked immunosorbent assay (ELISA), which is beneficial as it is less expensive [7,8]. The preferred treatment is tablet doxycycline 100 mg twice daily for seven days. Alternatively, an initial single oral dose of 500-1,000 mg of azithromycin, followed by 250-500 mg once daily is indicated in pregnancy and in other cases where tetracycline is contraindicated [9]. The disease course is complicated if left untreated and can become fatal.

Here we present two cases with atypical presentations of scrub typhus - hearing loss and atrial fibrillation - who reported to a tertiary care teaching hospital in rural Tamil Nadu, India. An awareness of these presentations can help clinicians have a better index of suspicion and initiate appropriate early treatment preventing morbidity and mortality.

## Case presentation

Case 1

**Figure 1:**
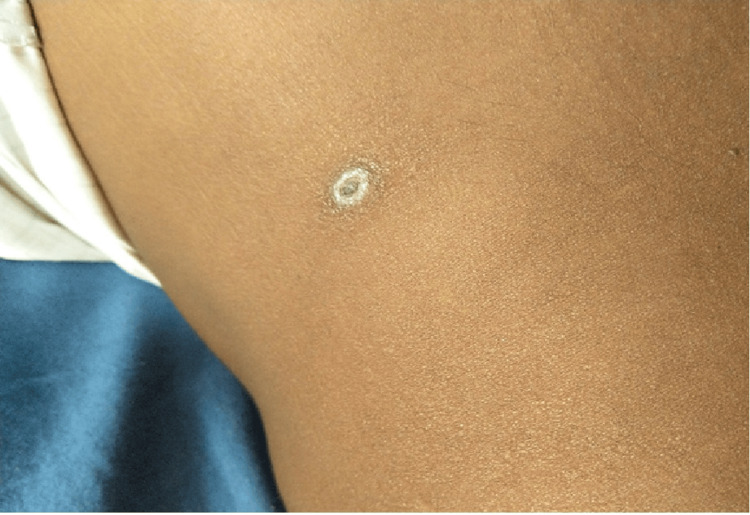
Eschar seen in the patient. The above eschar corresponds to a late stage in the evolution of eschar seen in scrub typhus.

A 46-year-old male, farmer by occupation, with no known comorbidities, presented to our hospital with complaints of fever for the past 10 days, which was high grade, intermittent, and not associated with chills or rigor, and generalized fatigue for the past five days. On detailed history taking, the patient gave a history of a small raised skin lesion over the left side of his abdominal wall, which later evolved into a black-crusted lesion. During the interrogation of history, we noticed the patient had difficulty hearing, and on further questioning, the patient said he had not been able to hear clearly for the past four to five days and that his hearing had been intact prior to the febrile illness. He had no history of ear pain, ear discharge, cough, running nose, nasal block, tinnitus, trauma, or recent exposure to any ototoxic drugs. On examination, the patient was febrile (101.6°F). He had a blood pressure of 100/70 mm Hg in the left upper limb, a pulse rate of 98 beats/minute, regular rhythm, respiratory rate of 16 breaths/minute, and oxygen saturation of 96% in room air. He had an eschar of size 1.5x1 cm in his left lumbar region (Figure 1).

**Figure 2:**
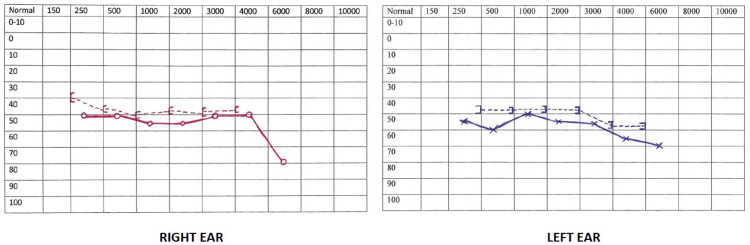
Pure-tone audiometry showing sensorineural hearing loss. Both the bone and air conduction thresholds are increased, and there is no air-bone gap. Horizontally, we have the frequencies in Hertz, and vertically, we have the hearing level of the patient in decibels. Box brackets indicate bone conduction (red: right ear; blue: left ear), red circles indicate air conduction in the right ear, and cross shapes indicate air conduction in the left ear.

On auscultation, the patient had bilateral wheeze, which settled with nebulizations. Investigations showed a hemoglobin level of 14.8 g/dL, a total white blood cell count of 8.7x10^9^/L (neutrophils 72.1%, lymphocytes 19.1%), and a platelet count of 190x10^9^/L (repeat platelet count was 140x10^9^/L). His renal function tests, liver function tests, blood sugar test, and urine analysis were within normal limits. ECG showed normal sinus rhythm with no ST-T changes. Scrub typhus Ig M ELISA was positive. Other workup done including leptospirosis IgM antibody, peripheral smear for malarial parasite, dengue serology, and blood and urine cultures were negative. Chest X-ray was normal. In the background of an eschar and positive serological test, a diagnosis of scrub typhus infection was made. The patient was started on tablet doxycycline 100 mg twice daily. An otorhinolaryngology opinion was obtained, pure-tone audiometry (Figure 2) was performed, and the patient was diagnosed with bilateral sudden sensorineural hearing loss and was advised tablet prednisolone 60 mg daily for one week.

The patient’s condition improved symptomatically, with resolution of wheeze, which was attributed due to mild bronchitis secondary to the scrub typhus infection. He had no oxygen requirement during the hospital stay. He was discharged and advised to continue tablet doxycycline 100 mg twice daily to complete a total duration of seven days and steroids for hearing loss. He was advised to review for repeat pure tone audiometry. During review after a month, his hearing loss had reversed.

Case 2

**Figure 3:**
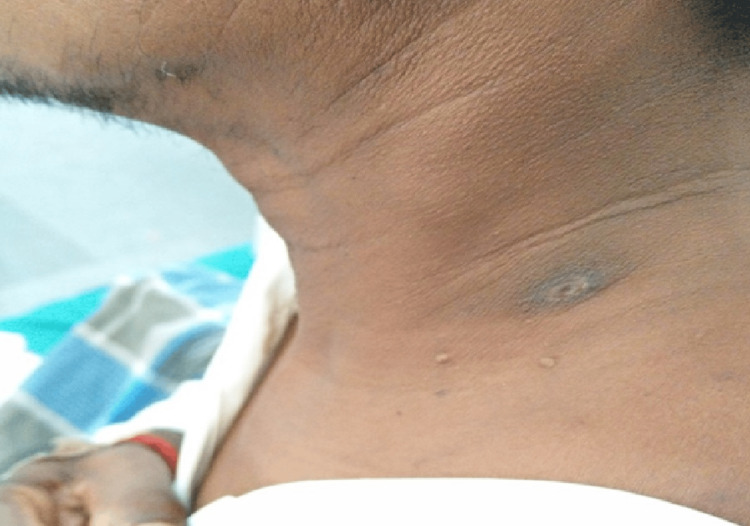
Eschar seen in the patient. The eschar seen corresponds to a later stage in the evolution of scrub typhus eschar wherein the crust has disappeared completely.

A 50-year-old male, farmer, presented to our hospital with complaints of fever for 10 days, which was high-grade, intermittent, and associated with chills and rigor, and 10 days of generalized body ache. The patient was a well-controlled diabetic, was not an alcoholic, and had no history of tobacco usage. On examination, the patient was febrile (101°F) and had a blood pressure of 120/70 mm Hg in the left upper limb, a pulse rate of 110 beats/minute, a respiratory rate of 22 breaths/minute, and oxygen saturation of 95% in room air; jugular venous pressure was not elevated. The patient had no pallor, cyanosis, or pedal edema. On careful and detailed examination, the patient was found to have an eschar of size 2x1.5 cm in the nape of his left neck (Figure 3).

**Figure 4:**
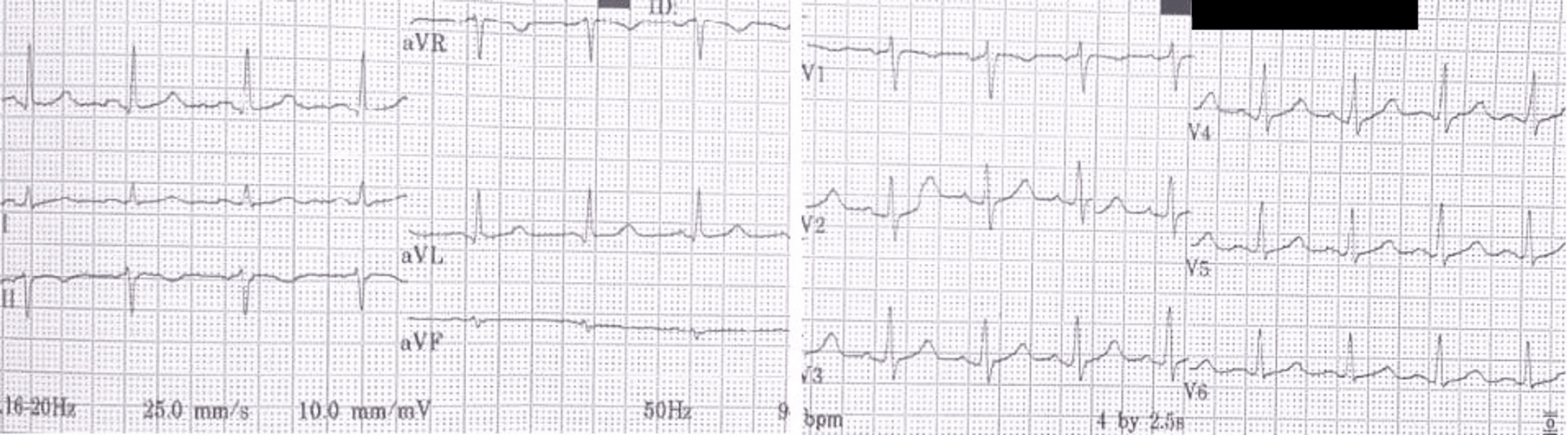
ECG at admission showing normal sinus rhythm

On auscultation, the patient had bilateral infrascapular crepitations and no cardiac murmur. Investigations showed a hemoglobin level of 9.5 g/dL, total white blood cell count of 13.1x10^9^/L (neutrophil 69.2%, lymphocyte 23.0%), and a platelet count of 116x10^9^/L. His renal function test, serum electrolytes, liver function test, and thyroid function test were normal. Scrub typhus Ig M ELISA was positive. Other workup done including leptospirosis IgM antibody, peripheral smear for malarial parasite, dengue serology, and blood and urine cultures were negative. His ECG was initially normal at admission (Figure 4).

**Figure 5:**
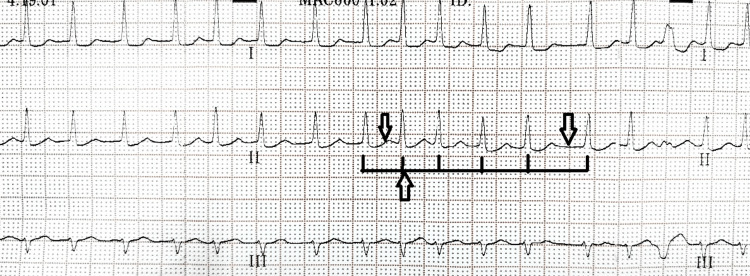
ECG during episode of atrial fibrillation. Arrows point toward irregular R-R interval and no discernable “P” waves.

On the next day, in view of the patient’s complaint of chest tightness and an irregularly irregular pulse clinically, repeat ECG was performed, which showed atrial fibrillation (Figure 5).

**Figure 6:**
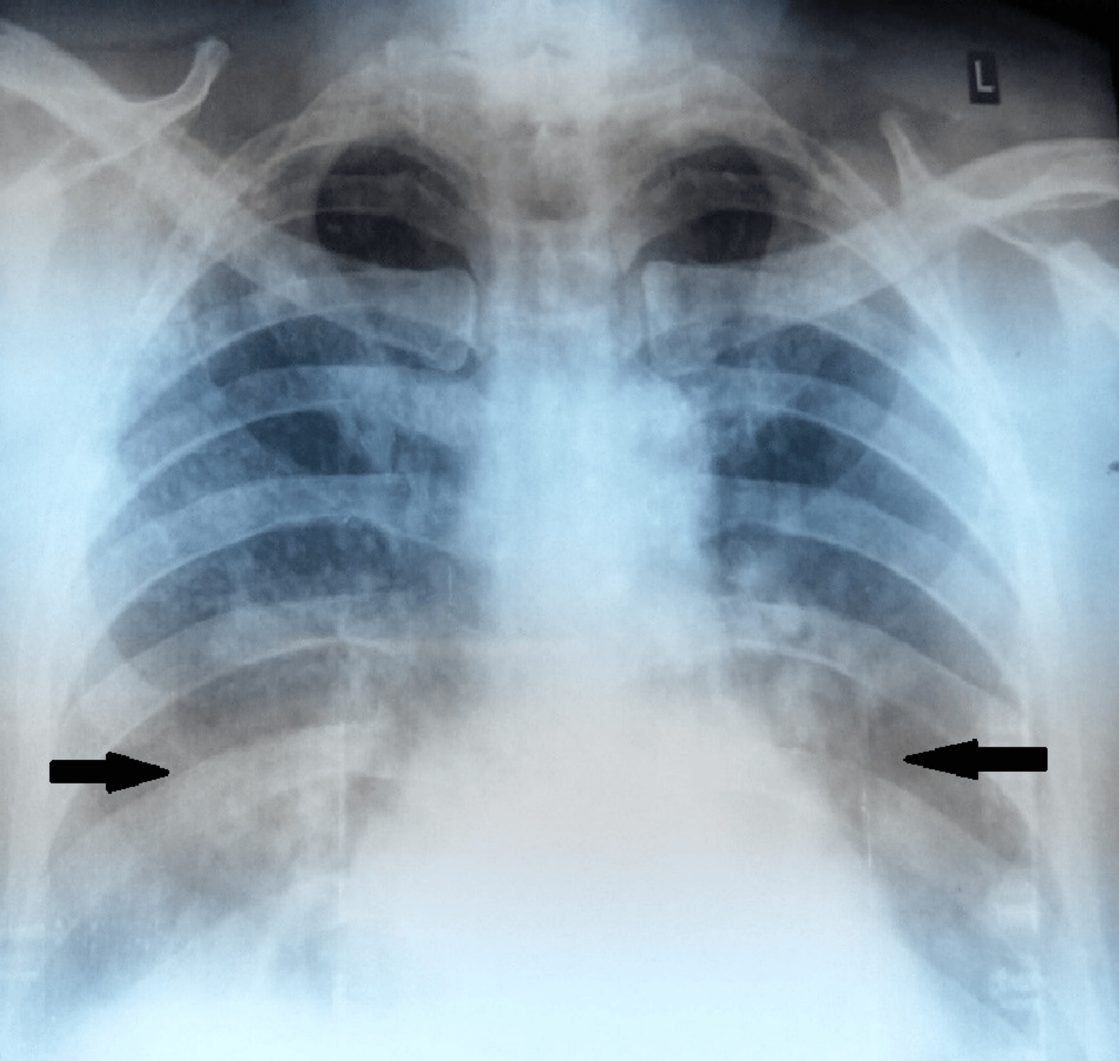
Chest X-ray of the patient, with arrows showing bilateral infiltrates.

Chest X-ray showed bilateral airspace opacities (Figure 6).

Echocardiography and 24-hour Holter monitoring were performed, which were normal after the episode of atrial fibrillation. The patient was started on tablet doxycycline 100 mg twice daily, and treatment was continued based on confirmation by the serology reports for seven days. The patient’s cough had improved, no further atrial fibrillation episodes were noted, and he was discharged afebrile.

## Discussion

A diagnosis of scrub typhus is made in most patients when they present with the classical clinical manifestations of fever, eschar, and seropositivity, as described in most literature. The presence of eschar has been reported in nearly 49% of patients in a study from South India, and the prevalence of thrombocytopenia was 79% [10].

Hearing loss has been reported to have a prevalence of 19%. It mostly appears in the second week after the onset of scrub typhus [11]. The mechanism behind hearing loss in scrub typhus infection could be either due to direct invasion of the pathogen into the central nervous system leading to vasculitis mediated damage to the cochlear component of vestibulocochlear nerve or due to vasculitis in the vasa vasorum of the cochlear nerve by a secondary immune mechanism [12,13]. Our patient presented with sudden onset hearing loss, which was of the sensorineural type according to pure-tone audiometry.

New-onset atrial fibrillation has been found to be associated with several infections and has a poor prognosis [14]. Studies have denoted that patients with scrub typhus who develop new-onset atrial fibrillation (1%) were more commonly above 60 years (87.2%) and had higher ICU hospitalization and mortality [15]. Atrial fibrillation could be triggered by the acute inflammation including myocardial inflammation, leading to electrical, functional, and structural remodeling [16]. ECG monitoring can be used for the identification of risk of adverse cardiac outcomes in patients with scrub typhus [17]. Other ECG abnormalities in scrub typhus infection include long QT, ischemic changes, arrhythmia, and atrioventricular conduction blocks. Moreover, QT prolongation occurs in approximately 20% of patients; hence, attention should be paid while prescribing drugs that can further prolong the QT interval in patients with scrub typhus [18]. Our patient had normal ECG at arrival and the next day developed paroxysmal atrial fibrillation from which he recovered spontaneously. He was only 50 years old and did not have much complications secondary to scrub typhus except for atrial fibrillation.

MODS has been found to occur in nearly 34% of patients with scrub typhus. Acute kidney injury occurs in 18% and acute hepatic dysfunction occurs in 87% of patients [10]. Respiratory organ dysfunction is the predominant finding in patients with MODS (96.6%). Despite the MODS, patients were found to have a good survival of 76% when treated [19].

## Conclusions

Scrub typhus is a tropical disease that often presents with varied manifestations. However, it is simple to treat, and patients show a predictable and good response to treatment. The infection sometimes masquerades and then it becomes difficult to diagnose; hence, diagnosis may be missed when the patient presents with atypical manifestations. Also, diagnostic tests in most cases are not feasible in rural areas due to either lack of availability or the cost factor. In such situations, a knowledge of the atypical manifestations can help in early diagnosis and treatment of this easily treatable disease.
